# Data of the interaction mindset questionnaire: An initial exploration

**DOI:** 10.1016/j.dib.2020.106000

**Published:** 2020-07-05

**Authors:** Masatoshi Sato, Kim McDonough, Juan Carlos Oyanedel

**Affiliations:** a Universidad Andres Bello, Department of English, 700 Fernandez Concha, Santiago, Chile; b Concordia University, Canada; c Universidad Andres Bello, Faculty of Education and Social Sciences

**Keywords:** Interaction mindset, Collaborative interaction, Peer feedback, Learner psychology, Task-based interaction

## Abstract

The survey data derives from a newly-developed questionnaire of interaction mindset. Interaction mindset pertains to second language (L2) learners’ disposition towards the task and/or an interlocutor prior to and/or during task-based interaction [1]. The theoretical model is consisted of five factors: (a) peer interaction; (b) collaboration; (c) form-orientation; (d) provision of peer feedback; and (e) reception of peer feedback. In the larger study (“Predicting L2 learners’ noticing of L2 errors: Proficiency, language analytical ability, and interaction mindset” [2]), the questionnaire results were used as predictor variables of L2 learners’ attention to language form. The current dataset contains responses from 102 L2 learners in university-level English classes. In addition to the descriptive statistics of the questionnaire, the current article reports on the results from structural equation modeling explaining the unique contributions of the five factors to the construct of interaction mindset. The model is visually depicted with a figure using AMOS. The model shows the questionnaire's potential in examining L2 learners’ affective variables that may influence the learners’ cognitive and behavioural engagement patterns. The entire dataset is included in an Excel file (.xlsx) and the original questionnaire is included as a supplementary file.

**Specifications Table**

**Table:** 

**Subject**	Linguistics and Language
**Specific subject area**	Instructed second language acquisition aims to examine the impact of second language teaching on learners’ second language development.
**Type of data**	Table Figure
**How data were acquired**	Survey (hardcopy) SEM (SPSS) AMOS
**Data format**	Raw Analyzed
**Parameters for data collection**	The survey was designed to target second language learners regardless of their target language or age.
**Description of data collection**	The sampling was done at a private university in Santiago, Chile. The respondents were pre-service English teachers belonging to a teacher training program. Originally, all students in the program from four year-levels were included (*N* = 135). After removing incomplete responses, the final dataset contained 102 responses.
**Data source location**	Institution: Universidad Andres Bello City/Town/Region: Santiago Country: Chile Latitude and longitude (and GPS coordinates) for collected samples/data: −33.375892, −70.504542
**Data accessibility**	With the article
**Related research article**	M. Sato, K. McDonough, Predicting L2 learners’ noticing of L2 errors: Proficiency, language analytical ability, and interaction mindset, System. In Press. https://doi.org/10.1016/j.system.2020.102301

## Value of the data

• This is a dataset of the interaction mindset questionnaire.
• The data can be useful for second language researchers who are interested in learner psychology and peer interaction.
• The data inform future research examining second language learners’ interaction mindset and its predictive power of interactional behavior.
• The data serve as the initial attempt to validate the five-factor construct of interaction mindset.
• The data can be compared with future research using the questionnaire with different groups of second language learners.

## 1 Data description

**Fig. 1:**
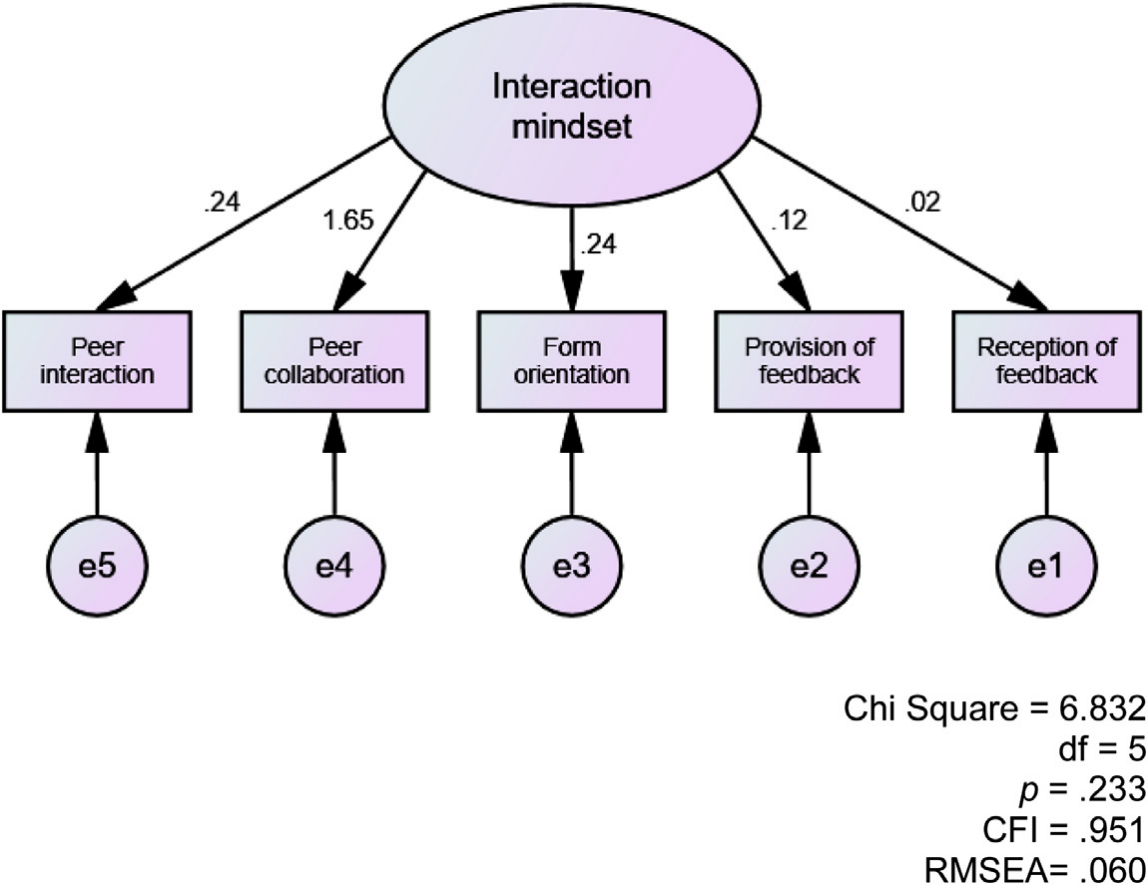
Structure of interaction mindset.

The survey responses included 102 university-level L2 learners of English. The survey was administered in December 2017. The participants answered the paper-and-pencil questionnaire in the classroom. The survey was conducted in English. The survey took a form of a 6-point Likert scale (1 = strongly disagree; 2 = mostly disagree; 3 = disagree; 4= agree; 5 = mostly agree; 6 = strongly agree). Data was collected with pre-coded alternatives. There was no missing data and no imputation procedures were used. The following items were reverse-coded: Items 3, 7, 12, 13, 15, and 16. In this article, first, the descriptive statistics of the data are presented (Table 1). Second, the unique contributions of the five factors to the construct of interaction mindset are presented through a confirmatory factor analysis (Fig. 1).

**Table 1:** Descriptive Statistics of Interaction Mindset Questionnaire Results (*N* = 102).

		M	SD	Range
Factor 1: Peer interaction (α = 0.69)				
Item 1:	I enjoy communicative activities with my classmates.	5.17	0.89	2.00–6.00
Item 2:	Communicative activities with my classmates are helpful for improving my English.	5.47	0.84	2.00–6.00
Item 3:	I don't like doing group work in my English classes. (RS)	4.17	0.89	1.00–6.00
Item 4:	I like talking to my classmates in English in class.	4.96	0.93	3.00–6.00
	*subtotal*	*4.94*	*0.64*	*2.50–6.00*
Factor 2: Collaboration (α = 0.68)				
Item 5:	I collaborate with my classmates to practice and learn English.	4.57	0.91	2.00–6.00
Item 6:	I try to help my classmates improve their English.	4.58	0.95	2.00–6.00
Item 7:	I don't care about my classmates’ improvement of English. (RS)	4.65	1.29	1.00–6.00
Item 8:	Working collaboratively is necessary to improve my English.	4.95	1.01	2.00–6.00
	*subtotal*	*4.69*	*0.75*	*2.25–6.00*
Factor 3: Form orientation (α = 0.68)				
Item 9:	When talking to my classmates, I tend to focus on their language use.	4.23	0.85	2.00–6.00
Item 10:	It's important to me to pay attention to how correctly my classmates speak English.	4.65	0.78	3.00–6.00
Item 11:	I tend to notice English mistakes when my classmates are talking to me.	4.54	1.07	2.00–6.00
	*subtotal*	*4.47*	*0.71*	*3.00–6.00*
Factor 4: Provision of peer feedback (α = 0.66)				
Item 12:	I think it's rude to correct my classmates’ English mistakes. (RS)	3.24	1.19	1.00–6.00
Item 13:	I think correcting my classmates’ English mistakes interrupts communication. (RS)	3.56	0.99	1.00–6.00
Item 14:	I feel comfortable correcting my classmates’ English mistakes.	3.80	1.29	1.00–6.00
	*subtotal*	*3.53*	*0.90*	*1.00–5.67*
Factor 5: Reception of peer feedback (α = 0.63)				
Item 15:	I feel embarrassed when my classmates correct my English mistakes. (RS)	4.15	0.92	1.00–6.00
Item 16:	I don't think my classmates’ corrections of my mistakes are accurate. (RS)	5.18	0.78	3.00–6.00
Item 17:	Students should correct each other's English mistakes.	4.81	0.99	2.00–6.00
	*subtotal*	*4.71*	*0.68*	*2.67–5.67*

*Note.* RS = reverse score.

Table 1 presents the descriptive statistics of each item (means, standard deviations, and ranges) as well as the subtotals for the five factors. In addition, the internal consistencies, calculated with Cronbach's alpha, are included. The first factor was named *peer interaction* because the four items tapped into the learners’ general attitudes towards pair/group work in the classroom. The second factor narrowed the general perceptions of peer interaction down to *collaboration*. Factor 3 included three items eliciting a learner's belief about how much attention they pay to language form; hence, this factor was named *form-orientation*. Factors 4 and 5 related to peer feedback specifically. While Factor 4 pertained to perceptions of *provision of peer feedback*, Factor 5 tapped into perceptions related to *reception of peer feedback*.

In exploring the construct of interaction mindset, the following steps were followed. First, Cronbach's alphas for each of the five factors were calculated using IBM SPSS Statistics 24. Subsequently, four items (i.e., Items 3, 7, 14, 17) were removed based on the information from “alpha score if removed.” Second, for each corrected factor, an exploratory factor analysis (principal components analysis) was run, with each dimension loading on a single factor. Bartlett scores were calculated and saved for each factor. Finally, the Bartlett scores were entered in structural equation modeling using IBM SPSS AMOS 24. A confirmatory factor analysis using a maximum likelihood estimation was used.

Fig. 1 depicts the structure of interaction mindset whereby the five factors contribute to the latent variable in unique manners. The results show a good fit with a CFI of 0.951 and a RMSEA of 0.060. The model shows that the learners’ perception of *peer collaboration* carries the most significant contribution, followed by *peer interaction* and *form-orientation*. Perceptions related to peer feedback have been found to contribute to the construct of interaction mindset to a lesser extent and the learners’ perceptions related to *reception of peer feedback* were the least related factor to interaction mindset.

## 2 Experimental design, materials, and methods

The participants were enrolled in an undergraduate degree program in English Education at a large private university in Santiago, Chile. At first, all students belonging to the program were included in the sample (*N* = 135). The researchers approached the participants by (a) getting an approval from the director of the program and the instructors, and (b) visiting English classes to include all potential participants. Subsequently, students who were absent from the classes when the data was collected were removed from the original pool. All students who were present in the classes agreed to participate in the study and the resulting sample size was 110. From the collected dataset, incomplete responses were removed, resulting in the final sample size of 102 (61 females and 41 males).

The participants belonged to different years of the program (Year 1: *n* = 26; Year 2: *n* = 29; Year 3: *n* = 36; Year 4: *n* = 11) and thus represented learners at different stages of L2 learning. Their ages ranged from 18 to 29, with a mean age of 21.8 (*SD* = 2.3). The program's curriculum aimed at A1 to C1 levels in the Common European Framework of Reference, depending on the year levels. The researchers visited six classes to collect data during the regular class periods. The data was collected via hardcopies of the instruments (pen and paper).

Based on the theoretical model of interaction mindset [1], a questionnaire was developed. Two areas in the literature were consulted. First, we explored the literature of second language (L2) learner psychology (e.g., L2 motivation, willingness to communicate, task attitudes, etc.) that has shown a link between learner psychology and L2 processing during interaction (e.g., [3], [4], [5]). Second, we surveyed interview questions in socially-oriented studies that have reported that affective/social factors influence the ways in which L2 learners engage with their partners and/or the task (e.g., [6], [7], [8]).

Based on the above literature, the initial questionnaire included 24 items with a 6-point Likert scale (1 = strongly disagree; 6 = strongly agree). The participants’ responses were submitted to an exploratory factor analysis. During the screening process, seven items were removed. The first criterion for removal was multicollinearity (correlation coefficients higher than 0.80). The following two items were correlated at 0.82 and the first one was retained because it was deemed more theoretically aligned with the original model of interaction mindset: “I like talking to my classmates in English.”; “Working with my classmates is helpful for learning English.” The second criterion was low correlations below 0.30 (four items were removed). For instance, the item “Working with my classmates is helpful for learning content (e.g., British culture)” did not corelate with any other items with a coefficient larger than 0.28. Third, two more items were removed because they did not load to any group of items. The Barlett's test was significant (*p* < .001) assuring the factorability of the dataset. Another factor analysis was conducted with 17 items which yielded a five-factor solution that cumulatively accounted for 74.5% of the dataset. The data presented in the current article are based on the 17 items that were loaded to any factors.

## Ethics statement

This manuscript has not been published elsewhere or it is not under consideration for publication for other journals. The study was conducted by following Universidad Andres Bello's ethical standards as well as those by the government of Chile (Comisión Nacional de Investigación Científica y Tecnológica). The ethical approval was obtained from the university (Acta14–2016). Informed consents by the participants were obtained prior to the data collection.

## Declaration of Competing Interest

The authors declare that they have no known competing financial interests or personal relationships which have, or could be perceived to have, influenced the work reported in this article.

## Appendix Supplementary materials

Image, application 1

Image, application 2
