# Supplementary material for: Data of the interaction mindset questionnaire: An initial exploration
Source: Data Brief. 2020 Jul 5;31:106000. doi: 10.1016/j.dib.2020.106000 (PMC7363661; doi:10.1016/j.dib.2020.106000)
Supplement: Supplementary file 1 [file mmc1.docx]

Interaction Mindset Questionnaire

Factor 1: Peer interaction (4 items)

- I enjoy communicative activities with my classmates.
- Communicative activities with my classmates are helpful for improving my English.
- I don’t like doing group work in my English classes. (reverse score)
- I like talking to my classmates in English in class.

Factor 2: Peer collaboration (4 items)

- I collaborate with my classmates to practice and learn English.
- I try to help my classmates improve their English.
- I don’t care about my classmates’ improvement of English. (reverse score)
- Working collaboratively is necessary to improve my English.

Factor 3: Form orientation (3 items)

- When talking to my classmates, I tend to focus on their language use.
- It’s important to me to pay attention to how correctly my classmates speak English.
- I tend to notice English mistakes when my classmates are talking to me.

Factor 4: Provision of peer feedback (3 items)

- I think it’s rude to correct my classmates’ English mistakes. (reverse score)
- I think correcting my classmates’ English mistakes interrupts communication. (reverse score)
- I feel comfortable correcting my classmates’ English mistakes.

Factor 5: Reception of peer feedback (3 items)

- I feel embarrassed when my classmates correct my English mistakes. (reverse score)
- I don’t think my classmates’ corrections of my mistakes are accurate. (reverse score)
- Students should correct each other’s English mistakes.

The original questionnaire (24 items before factor analysis)

- I enjoy communicative activities with my classmates.
- Communicative activities with my classmates are helpful for improving my English.
- I don’t like groupwork.
- I like talking to my classmates in English.
- Working with my classmates is helpful for learning English.
- Working with my classmates is helpful for learning content (e.g., British culture).
- When talking to my classmates, I focus on the meaning of what they are saying.
- When talking to my classmates, I tend to focus on their language use.
- I collaborate with my classmates to practice and learn English.
- I try to help my classmates improve their English.
- I don’t care about my classmates’ improvement of English.
- Working collaboratively is necessary to improve my English.
- Using correct English (grammar and pronunciation) is necessary for successful communication.
- It’s important to me to pay attention to how correctly my classmates speak English.
- It’s important to me to make sure I understand the meaning of my classmates’ comments.
- I tend to notice English mistakes when my classmates are talking to me.
- I think it’s rude to correct my classmates’ English mistakes.
- I feel embarrassed when my classmates correct my English mistakes.
- I think correcting my classmates’ English mistakes interrupts communication.
- I don’t believe my classmates’ corrections.
- Students should correct each other’s English mistakes.
- I feel comfortable correcting my classmates’ English mistakes.
- It is difficult for me to correct my classmates’ English mistakes.
- I don’t want to embarrass my classmates by correcting their English mistakes.
